# Molecular epidemiology of Methicillin-resistant *Staphylococcus aureus* in Africa: a systematic review

**DOI:** 10.3389/fmicb.2015.00348

**Published:** 2015-04-30

**Authors:** Shima M. Abdulgader, Adebayo O. Shittu, Mark P. Nicol, Mamadou Kaba

**Affiliations:** ^1^Division of Medical Microbiology, Department of Clinical Laboratory Sciences, Faculty of Health Sciences, University of Cape TownCape Town, South Africa; ^2^Department of Microbiology, Obafemi Awolowo UniversityIle-Ife, Nigeria; ^3^Institute of Infectious Disease and Molecular Medicine, Faculty of Health Sciences, University of Cape TownCape Town, South Africa; ^4^National Health Laboratory Service, Groote Schuur HospitalCape Town, South Africa

**Keywords:** *Staphylococcus aureus*, MRSA, molecular epidemiology, Africa, systematic review

## Abstract

Methicillin-resistant *Staphylococcus aureus* (MRSA) infections are a serious global problem, with considerable impact on patients and substantial health care costs. This systematic review provides an overview on the clonal diversity of MRSA, as well as the prevalence of Panton-Valentine leukocidin (PVL)-positive MRSA in Africa. A search on the molecular characterization of MRSA in Africa was conducted by two authors using predefined terms. We screened for articles published in English and French through to October 2014 from five electronic databases. A total of 57 eligible studies were identified. Thirty-four reports from 15 countries provided adequate genotyping data. CC5 is the predominant clonal complex in the healthcare setting in Africa. The hospital-associated MRSA ST239/ST241-III [3A] was identified in nine African countries. This clone was also described with SCC*mec* type IV [2B] in Algeria and Nigeria, and type V [5C] in Niger. In Africa, the European ST80-IV [2B] clone was limited to Algeria, Egypt and Tunisia. The clonal types ST22-IV [2B], ST36-II [2A], and ST612-IV [2B] were only reported in South Africa. No clear distinctions were observed between MRSA responsible for hospital and community infections. The community clones ST8-IV [2B] and ST88-IV [2B] were reported both in the hospital and community settings in Angola, Cameroon, Gabon, Ghana, Madagascar, Nigeria, and São Tomé and Príncipe. The proportion of PVL-positive MRSA carriage and/or infections ranged from 0.3 to 100% in humans. A number of pandemic clones were identified in Africa. Moreover, some MRSA clones are limited to specific countries or regions. We strongly advocate for more surveillance studies on MRSA in Africa.

## Introduction

Methicillin-resistant *Staphylococcus aureus* (MRSA) is a major public health concern and is responsible for both hospital- and community-associated infections worldwide (De Kraker et al., 2011; CDC, 2013; Falagas et al., 2013; Garza-González and Dowzicky, 2013; Lee et al., 2013; Chen and Huang, 2014). It is estimated that MRSA infections within the health care setting alone affected more than 150,000 patients annually in the European Union, with an additional cost of 380 million Euros (Köck et al., 2010). In the United States of America, 80,461 invasive MRSA infections and 11,285 related deaths occurred in 2011, and an estimated annual burden of between $1.4 billion and 13.8 billion was attributed to community-acquired MRSA (CDC, 2013; Lee et al., 2013). Besides, MRSA has been established as a pathogen for domestic animals and linked with livestock-associated infections (Verkade and Kluytmans, 2013).

Methicillin resistance is usually due to the *mecA* gene, borne on the staphylococcal cassette chromosome *mec* (SCC*mec*) that codes for a 78-kDa penicillin binding protein (PBP2a), with decreased affinity to methicillin and all beta-lactam antibiotics (Chambers, 1997). To date, eleven SCC*mec* types have been identified (IWG-SCC, 2009). Some cassettes, for example, SCC*mec* II (53 kb) and SCC*mec* III (67 kb), are large and possess mobile genetic elements (MGE), such as integrated plasmids (pUB110, pI258, and pT181) and transposons (e.g., Tn554) (Ito et al., 2001), and are frequently associated with hospital-acquired MRSA (Ma et al., 2002; Ito et al., 2004). In contrast, SCC*mec* IV (21–24 kb) and V (27 kb) are shorter elements, generally susceptible to non-beta-lactam antibiotics, and linked with community MRSA (Chambers and Deleo, 2010). However, the spread of various MRSA clones between the hospital and community settings has made the dichotomous ranking difficult (Deurenberg and Stobberingh, 2008). Recently, a variant *mecA* gene (named *mecC*) which is situated on an SCC*mec* XI element has been described (Shore et al., 2011). It has a higher relative affinity for oxacillin as compared with cefoxitin (Kim et al., 2012), and exhibits only 69% sequence similarity at the nucleotide level and 63% amino-acid identity to *mec*A/PBP2a (Paterson et al., 2014b). Furthermore, based on whole genome sequencing, mutations of the endogenous penicillin-binding proteins (PBP) 1, 2, and 3 in *mecA* and *mecC* negative strains have been postulated as a possible alternative mechanism for beta-lactam resistance in MRSA (Ba et al., 2014).

There is great interest in tracking, identifying and understanding the diversity of MRSA in various settings. Currently, the most widely used molecular techniques include *Staphylococcus* protein A gene typing (*spa*) and multilocus sequence typing (MLST). Studies (particularly using MLST) have provided evidence that a small set of lineages, clonal complex (CC)5, CC8, CC22, CC30, and CC45, are associated with most of the MRSA infections in hospitals (Stefani et al., 2012). Besides, a number of different geographically distinct lineages, CC1, CC8, CC30, and CC80, have also been associated with community MRSA infections (Chatterjee and Otto, 2013), while CC8 and CC30 have been identified as pandemic lineages both in the hospital and community setting (Chatterjee and Otto, 2013). Furthermore, regional clones have been described in Australia (sequence type [ST] 93) (Coombs et al., 2009), India (ST772) (D'Souza et al., 2010; Shambat et al., 2012), South Korea (ST72) (Kim et al., 2007), Taiwan and China (ST59) (Chen and Huang, 2014).

The distribution of MRSA clones in Africa is not well-described. Understanding the molecular epidemiology of MRSA in Africa is important as a recent review indicated that since the year 2000, the prevalence of MRSA appears to be increasing in many African countries and pose a visible threat to the continent (Falagas et al., 2013). Furthermore, there is evidence of the replacement of existing MRSA clones with different and new clonal types in a number of countries (Conceição et al., 2007; Aires-de-Sousa et al., 2008; Albrecht et al., 2011; Espadinha et al., 2013; Lim et al., 2013; Nimmo et al., 2013) but information on this trend is lacking in Africa. The occurrence and changes in clonal identities, and their geographic spread is important to understand the spread and evolution of MRSA.

The Panton-Valentine Leukocidin (PVL) is a two-component pore-forming toxin with cytolytic activity on defined cells of the immune system (neutrophils, macrophages and monocytes) (Löffler et al., 2010; Yoong and Torres, 2013). It is encoded by the *lukS-PV* and *lukF-PV* genes (Boakes et al., 2011), and PVL-producing *S. aureus* exhibit a propensity for causing mainly severe and often recurrent skin and soft tissue infections (Shallcross et al., 2013). In addition, PVL-positive MRSA are associated with community onset-pneumonia (Vandenesch et al., 2003). Although the PVL genes are mainly carried by community-associated MRSA (CA-MRSA) (Vandenesch et al., 2003), data from West and Central Africa showed that at least 40% of clinical methicillin-susceptible *S. aureus* (MSSA) isolates in this region are PVL-positive (Breurec et al., 2011a; Schaumburg et al., 2011; Shittu et al., 2011; Egyir et al., 2014a). Therefore, the acquisition of the *mecA* gene by PVL-positive MSSA and the possible dissemination of PVL-positive CA-MRSA could present a significant challenge in disease management and infection control in resource-limited countries in Africa.

This systematic review examined published literature on the molecular epidemiology of MRSA in Africa. By summarizing currently available data on the continent, our objective was to describe the distribution of MRSA clones, the prevalence of PVL-positive MRSA, and to highlight the need to develop more comprehensive surveillance and reporting systems for multidrug-resistant organisms such as MRSA in Africa.

## Methods

This systematic review was conducted in accordance with the preferred reporting items for systematic reviews and meta-analyses (PRISMA) guidelines (Moher et al., 2009).

### Literature search strategy

The relevant English and French articles available in five electronic databases (MEDLINE, EBSCOhost, ISI Web of knowledge, Scopus, and African Journals Online) were retrieved by two authors using predefined search terms (Table S1). The literature search was conducted until 31 October 2014.

### Eligible article identification

**Figure 1:**
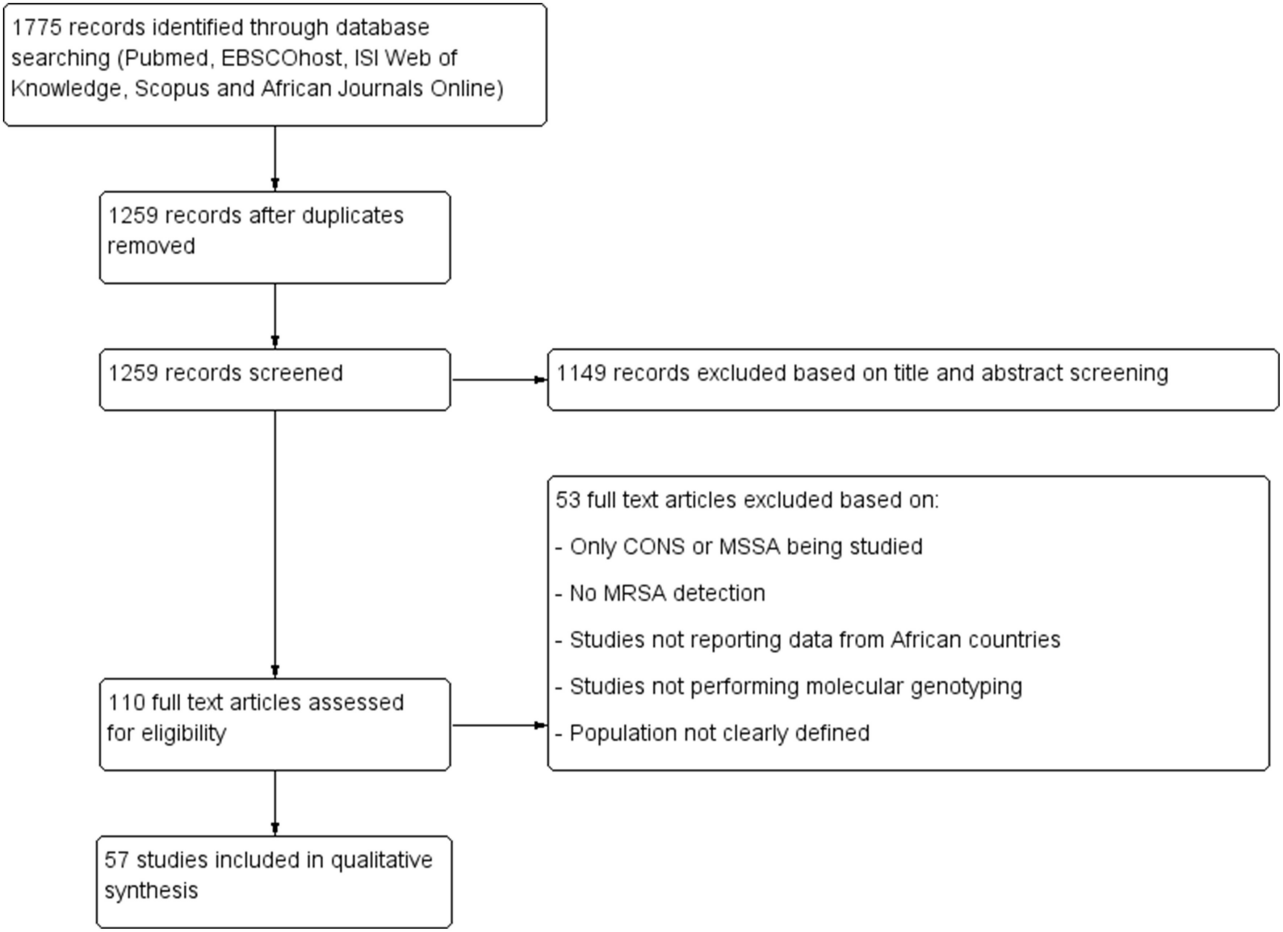
**Preferred reporting item for systematic reviews**. CONS, coagulase negative staphylococci; MSSA, methicillin susceptible *S. aureus;* MRSA, methicillin resistant *S. aureus.*

Figure 1 summarizes the study selection process. All duplicate articles were removed and data on MSSA as well as *in-vitro* studies were also excluded. The eligibility of published reports in this review was based primarily on polymerase chain reaction (PCR) detection of the *mecA* gene, and the use of at least one molecular tool for genotyping of MRSA strains (Table 1). In addition, worldwide surveys that covered African countries were also included. An MRSA clone was defined based on the combination of MLST sequence type (ST) and SCC*mec* typing data as previously reported (Okuma et al., 2002). The nomenclature of the SCC*mec* types was as proposed by the International Working Group on the Classification of Staphylococcal Cassette Chromosome Elements (IWG-SCC, 2009). SCC*mec* elements that could not be classified were indicated as non-typeable (SCC*mec*-NT). In this study, we categorized MRSA into various CCs according to the current eBURST scheme, Version 3 (accessed 30 October 2014) (eBURST, www.mlst.net, V3)^1^.

**Table 1:** **Characteristics of eligible articles that studied Methicillin resistant *Staphylococcus aureus***.

**Country**	**Study period**	**Study population (sample type)**	**No. of *S. aureus* isolates**	***S. aureus* molecular identification**	**No. of MRSA^¶^**	**Setting (no.)**	**Genotyping tools**						***PVL***	**Detection of genes**		**References**
							**SCC*mec***	***coa***	***agr***	***spa* typing**	**MLST**	**PFGE**		**Antibiotic resistance**	**Toxin/Virulence**	
**STUDIES CONDUCTED IN HUMANS**																
Algeria	2003–2004	Clinical samples from hospitals and community	614	–	204	HA (40)/CA (21)	✓	–	✓	–	✓	✓	✓	–	–	Ramdani-bouguessa et al., 2006
	2004–2007	Human infections (in- and-out patients)	65	–	23	NR	✓	–	–	✓	✓	–	✓	–	–	Bekkhoucha et al., 2009
	2005–2007	From military hospital (Pus, venous catheter, tracheal aspirates, lumbar puncture fluid, blood culture and urine)	NR	–	64	HA (50)/CA (14)	✓	–	–	–	–	–	✓	–	✓	Ouchenane et al., 2011, 2013
	2006–2007	Healthy and hospitalized individuals	221^α^ 52^β^	*gyrA* PCR	99^α^ 23^β^	HA (65)/CA (84)	✓	–	✓	✓	✓	NR	✓	–	✓	Antri et al., 2011
	2010–2011	Children and neonates (SSTI, bacteraemia, otitis and bone/joint infections)	129	–	25	HA (15)/CA (10)	✓	–	–	–	✓	✓	✓	–	–	Djoudi et al., 2013
Angola	2012	Nasal swabs from inpatients and HCW	117	–	68	NR	✓	–	✓	✓	✓	✓	✓	–	✓	Conceição et al., 2014
Egypt	2007–2008	Pus, sputum, wounds, abscess, blood, urine, and discharge	NR	–	21	CA (4)	✓	✓	✓	✓	✓	NR	✓	–	✓	Enany et al., 2010
	NR	SSTI and nasal swabs	38	–	18	CA (18)	✓	–	–	–	–	–	✓	–	–	Sobhy et al., 2012
	NR	Septic wounds, UTI and RTI (nasal swabs)	10	–	7	–	–	✓	–	✓	–	–	–	–	–	El-Jakee et al., 2011^*^
Gabon	2008–2010	asymptomatic carriers (nares, axillae, inguinal swabs) and patients (abscess, wound, blood and others)	217	*nuc* and 16S rRNA PCR	12	HA (6)/ CA (6)	✓	–	✓	✓	✓	–	✓	–	✓	Schaumburg et al., 2011; Ateba Ngoa et al., 2012
	2010–2013	swabs from *S. aureus* carrier mothers (nasal and mammillary) and their infants (Nasal and pharyngeal)	460	–	9	NR	✓	–	–	✓	✓	–	✓	–	✓	Schaumburg et al., 2014
	NR	Blood culture of one patient	1	–	1	NR	–	–	–	✓	✓	–	✓	–	✓	Huson et al., 2014
Ghana	2011–2012	In-patients and hospital staff	105	*spa* gene PCR	6	NR	✓	–	–	✓	✓	–	✓	–	–	Egyir et al., 2013
	2010–2012	SSTI and blood samples from six hospitals	308	–	9	NR	✓	–	–	✓	✓	–	✓	–	–	Egyir et al., 2014a
	2011–2012	Nasal swabs from apparently healthy carriers	124	–	2	HA (2)	✓	–	–	✓	✓	–	✓	–	–	Egyir et al., 2014b
Kenya	2005–2007	In and out-patients with SSTI boil, abscess, cellulitis and ulcer	84	–	69	NR	✓	–	–	–	–	–	✓	–	–	Maina et al., 2013
	2011	Nasal and axillary skin swabs from hospitalized patients	85	–	6	NR	✓	–	–	✓	✓	–	✓	✓	✓	Aiken et al., 2014
Libya	2009–2010	Nasal swabs from in-patient children, their mothers, out-patient children and HCW	758	–	70	HA (12) CA (6)	–	–	–	–	–	✓	✓	–	–	Al-haddad et al., 2014
Mali	2005	Asymptomatic nasal carriers	88	–	1	CA (1)	–	–	–	–	✓	–	–	–	–	Ruimy et al., 2008
Mozambique	2010–2011	Post-operative, burn wound infections, skin and soft tissue abscesses	99	–	9	HA (8), CA (1)	–	–	–	✓	–	–	✓	✓	–	Van der Meeren et al., 2014
Nigeria	1998–2002	Wounds, aspirate, amniotic fluid	276	–	4	NR	✓	–	–	–	✓	✓	–	–	–	Adesida et al., 2005
	2002–2004	Wound samples, blood cultures, urine, otitis media and ocular related infections	200	–	3	NR	✓	–	–	–	–	✓	–	–	–	Shittu and Lin, 2006b
	2007–2012	Clinical specimen	150	–	12	NR	✓	–	–	–	✓	–	✓	–	✓	Okon et al., 2009, 2014
	2007	Surgical and pediatric patients wound samples, corneal, conjunctival, auricular, genital and nasal swabs	346	–	70	HA (42), CA (28)	✓	–	✓	✓	✓	–	✓	–	✓	Ghebremedhin et al., 2009
	2008–2010	HIV-positive and healthy individuals (nasal swabs)	202	–	26	NR	–	✓	–	✓	✓	–	✓	–	–	Olalekan et al., 2012
	2009	Wound infections, semen, UTI, chronic ulcer, conjunctivitis, throat infections	68	–	11	NR	✓	–	–	✓	✓	–	✓	✓	–	Shittu et al., 2011
	2009–2011	Patients and carriers	62	–	22	NR	✓	–	–	–	✓	–	✓	–	–	Raji et al., 2013
	2010	Clinical samples from patients with burns, septicaemia, wound infections, osteomyelitis, bronchitis and GIT	51	*tuf* gene PCR	15	NR	✓	–	–	✓	✓	–	✓	✓	–	Shittu et al., 2012
	NR	Urine, blood and aspirates, wound, eye and ear, urethral and endocervical swab	116	–	48	HA (40), CA (8)	✓	–	–	–	–	–	✓	–	✓	Terry Alli et al., 2012
South Africa	2001–2003	Wound samples, sputum, otitis media and blood culture	227	*nuc* gene PCR	61	NR	✓	✓	–	–	–	✓	–	✓	–	Shittu and Lin, 2006a; Shittu et al., 2007
	2001–2003	Isolates from 16 laboratories in KZN	241	–	24	NR	✓	–	–	–	✓	–	–	–	–	Essa et al., 2009
	2001–2003	Wounds, sputum, otitis media, urine and blood culture	NR	–	61	NR	✓	–	–	✓	✓	✓	✓	–	–	Shittu et al., 2009
	2005–2006	Bacteraemia, SSTI, urine, catheter tip, cerebrospinal and drainage fluids	NR	–	320	HA	✓	–	–	✓	✓	✓	✓	–	✓	Moodley et al., 2010
	2006–2007	Clinical samples	NR	–	97	HA (79), CA (4)	✓	–	–	✓	–	–	✓	–	–	Makgotlho et al., 2009
	2007–2008 2007–2011	Pus and pus swabs, urine, blood, RTS and CVCT	NR	–	100	CA (10)	✓	–	–	✓	✓	✓	✓	–	✓	Jansen van Rensburg et al., 2011, 2012
	2009–2010	A wide range of clinical specimens mostly SSTI	367	–	56	NR	✓	–	✓	✓	✓	✓	✓	–	–	Oosthuysen et al., 2014
São Tome and Príncipe	2010–2012	Patients and healthy carriers	52	–	14	NR	✓	–	✓	✓	✓	✓	✓	–	✓	Conceição et al., 2013
Tanzania	2008	Wound, nasal swab and pus	160	–	24	HA	–	–	–	✓	✓	–	✓	–	–	Moremi et al., 2012
	2010	Apparently healthy children under 5 years (nasal swabs)	114	*nuc* gene PCR	12	CA	–	–	–	–	–	✓	–	–	–	Moyo et al., 2014
Tunisia	1998–2007	Clinical specimens from neutropenic patients	72	*nuc* gene PCR	13	HA (13)	✓	–	–	–	–	✓	–	–	–	Bouchami et al., 2009
	2002	Patients who developed MRSA infections	NR	–	6	HA (6)	–	–	–	–	–	✓	–	–	–	Ben Ayed et al., 2010
	2003–2004	Pus, blood, pleural fluid, venous catheter	NR	–	72	NR	✓	–	✓	–	–	–	✓	–	✓	Ben Nejma et al., 2006
	2003–2004	Pathological samples from different wards	147	–	19	NR	–	–	–	–	–	✓	–	–	–	Ben Saida et al., 2005
	2003–2005	Pus and associated with cutaneous infections	NR	–	64	CA (64)	✓	–	✓	✓	✓	✓	✓	–	✓	Ben Nejma et al., 2013
	2004	Cutaneous pus, blood cultures, urine and puncture fluids	NR	–	34	HA (32), CA (2)	✓	–	–	–	–	–	–	–	–	Ben Jomaa-Jemili et al., 2006
	2004–2005	Cutaneous pus, RTS, urine, blood culture,	475	–	57	NR	–	–	✓	–	–	–	–	–	–	Ben Ayed et al., 2006
	2004–2008	Samples from hospitals and community	NR	–	69	HA (41), CA (28)	✓	–	✓	✓	✓	–	✓	–	–	Ben Jomàa-Jemili et al., 2013
	2006–2008	Children with CA invasive infections bacteraemia and osteomyelitis	36	–	8	CA (8)	✓	–	✓	✓	✓	✓	✓	–	–	Kechrid et al., 2011
	2007	Pus and skin infections	NR	–	11	CA (11)	✓	–	✓	✓	✓	✓	✓	–	–	Ben Nejma et al., 2009
	2008	Pus and blood culture (case report)	2	–	2	NR	✓	–	✓	✓	✓	–	✓	–	✓	Zribi et al., 2011
	2008–2009	Humans in contact with animals	55	–	1	CA (1)	✓	–	✓	✓	✓	–	✓	✓	✓	Ben Slama et al., 2011
Uganda	2009–2010	Swabs from patients, HCW and from hospital environment (sinks, door handles, surgical trays, bed and table surfaces)	41	–	41	NR	✓	–	–	–	–	–	✓	✓	✓	Kateete et al., 2011
	2011–2012	SSI	64	*nuc* gene PCR	24	NR	✓	–	–	✓	–	–	✓	–	–	Seni et al., 2013
Multicenter^#^	2007–2008	SSTI, bacteraemia/septicaemia, urine, wounds osteomyelitis and myositis	NR	–	86	CA (9), HA (77)	✓	–	✓	✓	✓	–	✓	–	✓	Breurec et al., 2011b
Multicentre^✖^	2004–2005	Uncomplicated skin infections	292	–	105	HA (3)	✓	–	–	–	✓	✓	✓	–	–	Goering et al., 2008
**STUDIES CONDUCTED IN ANIMALS**																
Egypt	NR	Cows and buffaloes milk, cattle septic wounds	9	–	5	NR	–	✓	–	✓	–	–	–	–	–	El-Jakee et al., 2011^*^
Senegal	2009–2011	Pigs (nasal swabs)	73	–	6	NR	✓	–	–	✓	✓	–	✓	–	✓	Fall et al., 2012
Tunisia	2010	Healthy sheep (nasal swabs)	73	–	5	CA (6)	✓	–	✓	✓	✓	✓	–	–	✓	Gharsa et al., 2012

agr, Accessory gene regulator; CA, Community-acquired methicillin-resistant S. aureus; coa, Coagulase gene; CVCT, Central venous catheter tips; GIT, Genital tract infections; gyrA, DNA gyrase gene; HA, Hospital-acquired methicillin resistant S. aureus; HCW, Health care workers; HIV, Human immunodeficiency virus; KZN, KwaZulu-Natal province; MLST, Multilocus locus sequence typing; MRSA, Methicillin-resistant Staphylococcus aureus; No., Number of isolates; NR, Not reported; nuc, Thermonuclease gene; PCR, Polymerase chain reaction; PFGE, Pulsed-field gel electrophoresis; PVL, Panton-Valentine-Leukocidin genes; SCCmec, Staphylococcal chromosomal cassette mec element; RTI, Respiratory tract infections; rRNA, Ribosomal ribonucleic acid; RTS, Respiratory tract specimens; spa, Staphylococcus aureus protein A gene; SSTI, Skin and soft tissue infections; SSI, Surgical site infections; tuf, Elongation factor tu; UTI, Urinary tract infections.

¶ MRSA as confirmed by mecA PCR;

✓, Test was conducted; −, Test was not conducted;

α Hospitalized individuals;

β Nasal carrier study;

# African multicenter study which included Cameroon, Madagascar, Morocco, Niger and Senegal;

✖ An international multicenter study which included only South Africa;

* Study was conducted in both animal and human host.

### Data extraction and synthesis

The relevant data were extracted from each of the articles as stated in Table 1. Separate articles that analyzed the same *S. aureus* isolates but answered different questions were considered as a single study.

### eBURST analysis

The relationship between the MRSA STs described in this review and other lineages reported world-wide was analyzed using the eBURST scheme. The allelic profiles were downloaded from the MLST website (http://saureus.mlst.net/) which included the African MRSA STs as well as 223 representative and randomly selected STs (from each CC) based on the differences in their allelic profiles. The minimum spanning tree was constructed by the goeBURST algorithm using the Phyloviz software v1.1 (http://www.phyloviz.net/).

## Results

### Literature search

The systematic search of the five electronic databases yielded 1775 articles (Figure 1). No additional studies were identified from AJOL. After the removal of duplicate studies and assessment of titles and abstracts, 110 full-text articles were screened, of which 57 studies were considered eligible for the qualitative analysis according to our inclusion criteria.

### Characteristics of the studies included in the systematic review

Most of the data analyzed were obtained from single center studies conducted mainly in five countries; Tunisia (*n* = 13), Nigeria (*n* = 9), South Africa (*n* = 7), Algeria (*n* = 5), and Egypt (*n* = 3) (Table 1). Multicenter studies were only reported in two articles (Goering et al., 2008; Breurec et al., 2011b), including a survey which comprised five African countries, Cameroon, Madagascar, Morocco, Niger and Senegal (Breurec et al., 2011b), and an inter-continental multicenter study, which included South Africa (Goering et al., 2008). Only three studies investigated the detection of MRSA in animals (Table 1).

In most of the reports included in this study, *S. aureus* was identified by phenotypic and culture characteristics, while molecular identification (16S rRNA, detection of the thermonuclease and the elongation factor tu - *nuc, tuf* - genes) was performed in only 12.3% (7/57). The screening for antibiotic resistance and toxin/virulence genes were carried out in seven and 22 studies, respectively (Table 1). Furthermore, all the eligible studies analyzed MRSA using at least one genotyping technique, and 59.6% (34/57) provided adequate genotyping data on MRSA clones from 15 African countries (Tables 1, 2). Studies included in this systematic review did not investigate on the *mecC* gene.

**Table 2:** **Methicillin resistant *Staphylococcus aureus* clones reported in 34 eligible studies**.

**Country**	**Clonal type ST-SCC*mec***	**Clonal complex**	***spa* type**	**PVL status**	***agr***	**References**
Algeria	ST80-IV [2B]	80	ND	+	III	Ramdani-bouguessa et al., 2006
	ST37-IVa [2B]	30	ND	+	III	
	ST239-III [3A]	5	ND	−	I	
	ST239-IVa [2B]	5	ND	−	I	
	ST241-III [3A]	5	ND	−	I	
	ST637-III [3A]	5	ND	−	I	
	ST5-IV, IVa [2B]	5	ND	+, −	II	
	ST635-IV [2B]	80	ND	−	III	
	ST636-NT	22	ND	−	I	
	ST80-IV [2B]	80	t044	+	ND	Bekkhoucha et al., 2009
	ST239-III [3A]	5	t037	−	ND	
	ST5-IVa [2B]	5	t311, t450	−	ND	
	ST88-NT	88	t188, t267	−	ND	
	ST80-IV [2B]	80	t044, t4143	+	III	Antri et al., 2011
	ST241-III [3A]	5	ND	−	III	
	ST8-V [5C]	5	ND	−	I	
	ST80-IVc [2B]	80	ND	+, −	ND	Djoudi et al., 2013
	ST39-II [2A]	30	ND	−	ND	
Angola	ST5-IVa [2B]	5	t105, t311, t11657	−	II	Conceição et al., 2014
	ST8-IVd, V [2B]	5	t104, t1774	−	I	
	ST72-V [5C]	5	t3092	−	I	
	ST88-IVa [2B]	88	t186, t325, t786,	−	III	
			t1951, t3869			
	ST5-V [5C]	5	t6065	−	II	
	ST2629-V [5C]	5	t6065	−	II	
	ST789-V [5C]	7	t091	−	II	
Cameroon	ST5-V [5C]	5	t311	+	II	Breurec et al., 2011b
	ST88-IV [2B]	88	t186	−	III	
	ST8-IV [2B]	5	t024, t121, t451	+	I	
	ST1289-V [5C]	88	t1339	+	III	
Egypt	ST80-IVc [2B]	80	t042, t044, t070, t983	+	III	Enany et al., 2010
	ST30-IVa [2B]	30	t251, t318	+	III	
	ST1010-X^a^	121	t159, t312	+	IV	
Gabon	ST8-IV [2B]	5	t121	+	I	Schaumburg et al., 2011; Ateba Ngoa et al., 2012
	ST88-IV [2B]	88	t186	−	III	
	ST5-IV [2B]	5	t653	−	II	
	ST5-IV [2B]	5	t653	−	ND	Schaumburg et al., 2014
	ST8-NT	5	t112, t121	+	ND	
	ST45-V [5C]	45	t437, t8860	−	ND	
	ST88-IV [2B]	88	t4195	−	ND	
Ghana	ST72-V [5C]	5	t537	−	ND	Egyir et al., 2013
	ST8-V [5C]	5	t064	−	ND	
	ST88-IV [2B]	88	t325, t1951, t2649	−	ND	
	ST72-V [5C]	5	t537	−	ND	Egyir et al., 2014b
	ST8-IV [2B]	5	t121	+	ND	
	ST239-III [3A]	5	t037	−	ND	
	ST250-I [1B]	5	t928	−	ND	
	ST2021-V [5C]	5	t024	−	ND	
	ST88-IV [2B]	88	t186	−	ND	
	ST789-IV [2B]	7	t547	+	ND	
	ST508-V [5C]	45	t5132	−	ND	Egyir et al., 2014a
Kenya	ST239-III [3A]	5	t037	−	ND	Aiken et al., 2014
Madagascar	ST8-IV [2B]	5	t121	+	I	Breurec et al., 2011b
	ST30-V [5C]	30	t4686	−	III	
	ST88-IV [2B]	88	t186	−	III	
Morocco	ST239, ST241-III [3A]	5	t037, t138	−	I	
	ST5-IV [2B]	5	t311	+	II	
Niger	ST239, ST241-III [3A]	5	t138	−	I	
	ST239, ST241-V [5C]	5	t037	−	I	
	ST88-IV [2B]	88	t186	−	III	
Nigeria	ST8-IV [2B]	5	ND	−	ND	Adesida et al., 2005
	ST88-IV [2B]	88	t186	+	III	Ghebremedhin et al., 2009
	ST241-IV [2B]	5	t037	−	I	
	ST250-I [1B]	5	t194, t292	−	I	
	ST241-III [3A]	5	t037	−	ND	Shittu et al., 2011
	ST8-V [5C]	5	t064	−	ND	
	ST8-V [5C]	5	t451	−	ND	
	ST94-IV [2B]	5	t008	−	ND	
	ST5-V [5C]	5	t002	−	ND	
	ST241-III [3A]	5	t037	−	ND	Shittu et al., 2012
	ST88-IV [2B]	88	t729, t1603	−	ND	
	ST37-III [3A]	30	t074	−	ND	
	ST39-II [2A]	30	t007	−	ND	
	ST8-V [5C], IV [2B], ST8-NT	5	t064	−	ND	
	ST152-NT	152	t4690	+	ND	
	ST1-V [5C]	5	ND	+	ND	Raji et al., 2013
	ST239-III[3A]_*mercury*_	5	ND	−	ND	
	ST5-II [2A]	5	ND	−	ND	
	ST8-V [5C]	5	ND	−	ND	
	ST247-I [1B]	5	ND	−	ND	
	ST772-V [5C]	5	ND	+	ND	
	ST88-IV [2B]	88	ND	−	ND	
	ST241-III [3A]	5	ND	−	ND	Okon et al., 2009
Senegal	ST5-IV [2B]^*^	5	t311	+	ND	Fall et al., 2012
	ST88-IV [2B]^*^	88	t3489	−	ND	
	ST239, ST241-III [3A]	5	t037, t138	−	I	Breurec et al., 2011b
	ST5-II [2A]	5	t311	+	II	
	ST5-IV [2B]	5	t311	+	II	
	ST88-IV [2B]	88	t168	−	III	
South Africa	ST5-IV [2B]	5	ND	ND	ND	Essa et al., 2009
	ST8-IV [2B]	5	ND	ND	ND	
	ST8-II [2A]	5	ND	ND	ND	
	ST239-III [3A]	5	ND	ND	ND	
	ST45-IV [2B]	45	ND	ND	ND	
	ST612-IV [2B]	5	ND	−	ND	Goering et al., 2008
	ST36-II [2A]	30	ND	−	ND	
	ST1173-IV [2B]	5	t064	−	ND	Shittu et al., 2009
	ST1338-IV [2B]	5	t064	−	ND	
	ST239-III [3A]	5	t037	−	ND	
	ST5-III [3A]	5	t045	−	ND	
	ST239-III [3A]	5	t037	−	ND	Moodley et al., 2010
	ST612-IV [2B]	5	t064	−	ND	
	ST5-I [1B]	5	t045	−	ND	
	ST22-IV [2B]	22	t032	−	ND	
	ST22-IV [2B]	22	t891	+	ND	
	ST36-II [2A]	30	t012	−	ND	
	ST239-III [3A]	5	t037	−	ND	Jansen van Rensburg et al., 2011
	ST5-I [1B]	5	t045	ND	ND	
	ST650-IV [2B]	5	t002	ND	ND	
	ST612-IV [2B]	5	t064, t1443, t2196	ND	ND	
	ST72-NT	5	t3092	ND	ND	
	ST22-IV [2B]	22	t032	ND	ND	
	ST36-II [2A]	30	t012, t021	ND	ND	
	ST5-I [1B]	5	t002	−	II	Oosthuysen et al., 2014
	ST8-V [5C]	5	t064	−	I	
	ST612-IV [2B]	5	t064	+	I	
	ST239-III [3A]	5	t021	−	I	
	ST22-V [5C]	22	t891	+	I	
	ST22-IV [2B]	22	t891	−	I	
	ST36-II [2A]	30	t021	−	III	
São Tome	ST5-IVa [2B]	5	t105	−	II	Conceição et al., 2013
and Príncipe	ST105-II [2A]	5	t002	−	II	
	ST8-V [5C]	5	t451	−	I	
	ST8-IV [2B]	5	t451	−	I	
	ST88-IVa [2B]	88	t186, t786	−	III	
Tunisia	ST80-IV [2B]	80	t044	+	III	Ben Nejma et al., 2009
	ST80-IV [2B]	80	t044	+	III	Ben Nejma et al., 2013
	ST728-IVc [2B]	80	t042, t044	+	III	Kechrid et al., 2011
	ST8-IVc [2B]	5	t062	+	II	
	ST80-IVc [2B]	80	t203	+	III	Ben Slama et al., 2011
	ST1-NT	5	t035	−	III	Ben Jomàa-Jemili et al., 2013
	ST247-I [1B]	5	t040	−	I	
	ST239-III [3A]	5	t003	−	I	
	ST241-III [3A]	5	t125	−	I	
	ST97-NT	5	t003	−	I	
	ST1819-I [1B]	5	NS	−	I	
	ST80-IVc [2B]	80	t070	+	III	
	ST2563-IVc [2B]	80	t070	+	III	
	ST1440-IVc [2B]	80	t070	+	III	
	ST80-IVc [2B]	80	t1021	−	II	
	ST80-IVc [2B]	80	ND	−	III	
	ST22-NT	22	t998	−	II	
	ST45-NT	45	ND	−	I	
	ST153-NT	80	NST	+	III	
	ST153-NT	80	t044	ND	III	Gharsa et al., 2012

agr, Accessory gene regulator; CC, Clonal complex; NT, Non typeable; ND, Not determined; NST, New spa type; PVL, Panton-Valentine Leukocidin genes; SCCmec, Staphylococcal chromosomal cassette mec element; ST, Sequence type; spa, Staphylococcus aureus protein A gene; X^a^, Unknown SCCmec type other than I, II, III, IV or V;

* Clones isolated from pigs;

+, PVL positive; −, PVL negative.

### Community- and hospital- acquired MRSA

Overall, 51% (29/57) of the eligible studies provided the potential source (hospital- or community-associated) of the MRSA strains. Only 17.5% (10/57) reported MRSA from community settings (Table 1). USA300 (ST8-IV [2B]) and other related sequence types were noted both in health care and community settings in nine African countries (Tables 1, 2). The “Brazilian/Hungarian clone” (ST239-III [3A]) was associated with hospital-acquired infections in nine countries (Tables 1, 2). Furthermore, the “West Australia MRSA-2” (ST88-IV [2B]) was reported in community- and hospital-acquired infections in several African countries (Table 2).

### Detection of panton-valentine leukocidin (PVL) genes

The screening for PVL-associated genes (lukF-PV and lukS-PV) was carried out in 44 studies, and the detection of PVL genes was only reported in 32 studies (Table 1). In animals, PVL-positive MRSA (ST5) was described in nasal samples of pigs from Senegal (Fall et al., 2012). In humans, the proportion of PVL-positive MRSA carriage and/or infections ranged from 0.3 to 100%. Studies from Algeria and Tunisia reported higher PVL prevalence while investigations from South Africa reported the lowest prevalence (Table 3). Overall, PVL-positive MRSA were more frequently reported with skin and soft tissue infections, and community-associated clones (Tables 1, 2). There was no report on the role of PVL in necrotizing pneumonia caused by MRSA in Africa.

**Table 3:** **Panton-Valentine Leukocidin prevalence as reported by the eligible studies with sample size of 30 or above**.

**Country**	**PVL positive (no. positive/total tested)**	**Prevalence (%)**	**References**
Algeria	46/61	75	Ramdani-bouguessa et al., 2006
	19/64	30	Ouchenane et al., 2011
	94/122	77	Antri et al., 2011
Kenya	14/69	20	Maina et al., 2013
Libya	10/35	29	Al-haddad et al., 2014
Nigeria	33/70	47	Ghebremedhin et al., 2009
South Africa	1/320	0.3	Moodley et al., 2010
	4/97	4	Makgotlho et al., 2009
	5/56	9	Oosthuysen et al., 2014
Tunisia	68/72	94	Ben Nejma et al., 2006
	64/64	100	Ben Nejma et al., 2013
	43/69	62	Ben Jomàa-Jemili et al., 2013
Uganda	30/41	73	Kateete et al., 2011
Multicenter^*^	20/86	23	Breurec et al., 2011b

* Multicenter study which included Cameroon, Madagascar, Morocco, Niger and Senegal.

PVL, Panton-Valentine Leukocidin; no., Number.

### MRSA clones reported in africa using the current eBURST scheme

**Figure 2:**
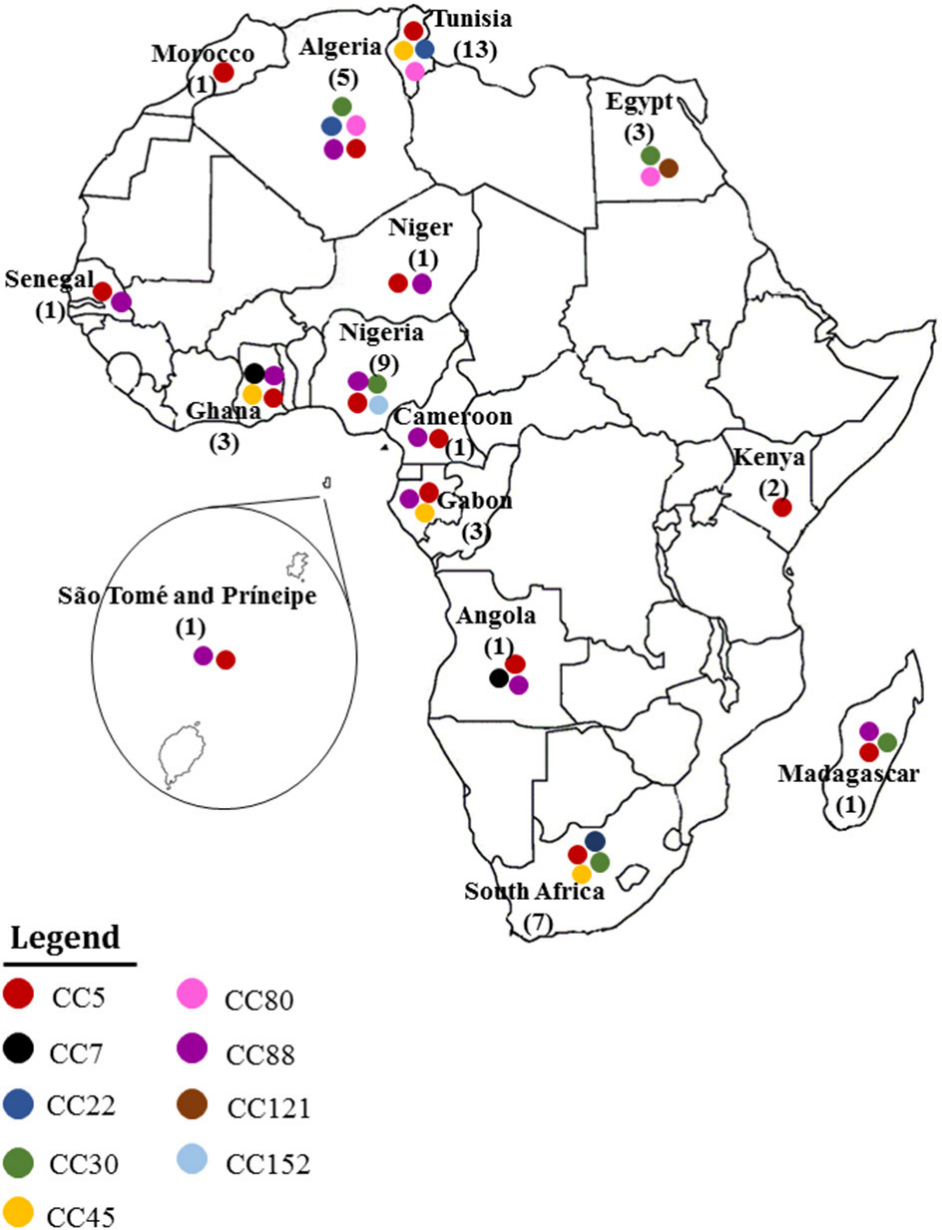
**MRSA clones reported in Africa**. Each clonal complex is annotated with a colored circle. The number of studies conducted in each country is also indicated.

**Figure 3:**
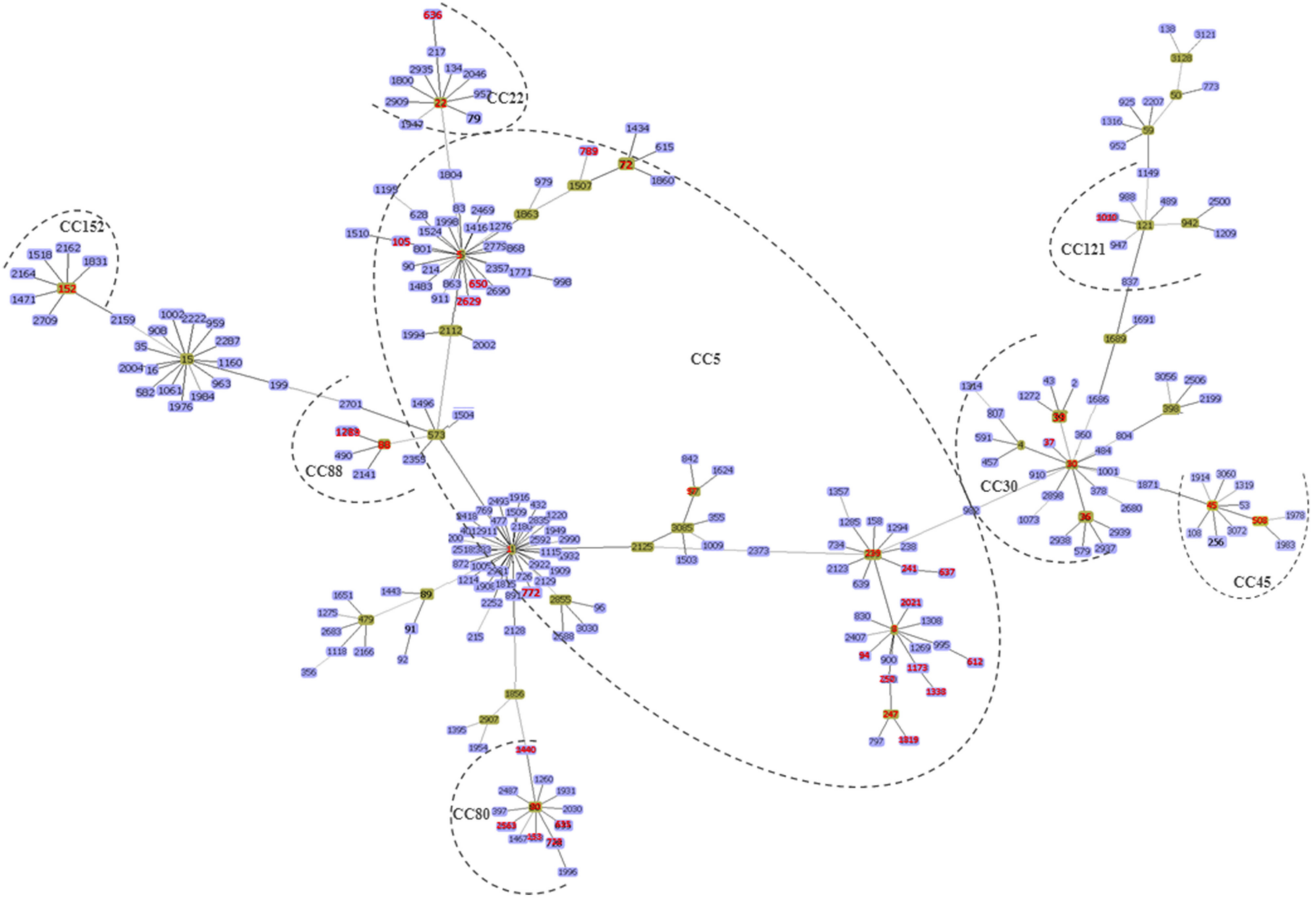
**The minimum spanning tree was constructed by the goeBURST algorithm using the Phyloviz software v1.1 (http://www.phyloviz.net/)**. The allelic profiles were downloaded from the MLST website (http://saureus.mlst.net/) which included the MRSA sequence types (STs) described in this review as well as 223 randomly selected STs (from each CC) based on the differences in their allelic profiles. The Group founder is colored in green and the related STs are in blue. The six main CCs described in this review are indicated by the dotted lines and the STs reported in Africa are indicated by the red color.

Figures 2, 3 summarize the MRSA clones identified in Africa based on MLST CCs. By the current eBURST scheme, six main CCs were identified: CC5, CC22, CC30, CC45, CC80, and CC88. In addition, a number of diverse *spa* types were identified among the MRSA clones in Africa (Table 2), but the distribution of *spa* types t042 and t044 (associated with CC80-IV [2B]) appear to be limited to three North African (Algeria, Egypt and Tunisia) countries (Table 2).

### Clonal complex 5

This clonal complex is considered the largest group based on the eBURST scheme (Figure 3). It was subdivided into three main clusters and designated as CC5-ST1, ST5, and ST8.

### MRSA CC5 with sequence type 1

This group was reported in Nigeria (Raji et al., 2013) and Tunisia (Ben Jomàa-Jemili et al., 2013). The clonal type included the PVL-positive ST1-V [5C] isolated from patients in a tertiary hospital in Nigeria (Raji et al., 2013), and the PVL-negative ST1 with a non-typeable SCC*mec* element (*spa* type t035 and *agr* type III) identified in Tunisia (Ben Jomàa-Jemili et al., 2013). In addition, an ST1 related sequence type (ST772-V [5C]), “the Bengal-Bay clone” has been described in Nigeria (Raji et al., 2013).

### MRSA CC5 with sequence type 5

This clone was documented in 14 studies and consisted of diverse SCC*mec* elements (Table 2). The ST5-I [1B]/III [3A] were identified from clinical samples in health care institutions in South Africa (Shittu et al., 2009; Moodley et al., 2010; Jansen van Rensburg et al., 2011; Oosthuysen et al., 2014). ST5-II [2A] has been described in Nigeria (Raji et al., 2013), and Senegal (Breurec et al., 2011b). ST5-IV [2B]-PVL-positive was the dominant clone in hospitalized patients with skin and soft tissue infections in Dakar, Senegal (Breurec et al., 2011b). In addition, ST5-IV [2B] was detected from nasal samples of pigs in the same geographical area (Fall et al., 2012). ST5-IV [2B] has also been identified in Algeria (Ramdani-bouguessa et al., 2006), Gabon (Schaumburg et al., 2011; Ateba Ngoa et al., 2012), Morocco (Breurec et al., 2011b), and South Africa (Essa et al., 2009), while the SCC*mec* IVa [2B] variant was recovered from hospitalized patients in Algeria (Ramdani-bouguessa et al., 2006; Bekkhoucha et al., 2009), Angola (Conceição et al., 2014), and São Tomé and Príncipe (Conceição et al., 2013). Moreover, ST5-IVa [2B] was reported from nasal samples of apparently healthy-hospital workers in Angola (Conceição et al., 2014). Other ST5 and related clones identified are ST5-V [5C] in Angola (Conceição et al., 2014), Cameroon (Breurec et al., 2011b), and Nigeria (Shittu et al., 2011), ST72-SCC*mec*-NT in South Africa (Jansen van Rensburg et al., 2011), ST72-V [5C] in Angola and Ghana (Egyir et al., 2013; Egyir et al., 2014b; Conceição et al., 2014), and ST105-II [2A] from a patient in São Tomé and Príncipe (Conceição et al., 2013). Furthermore, ST650-IV [2B] was detected from clinical samples in health care institutions in South Africa (Jansen van Rensburg et al., 2011). Finally, ST2629-V [5C] was described in Angola (Conceição et al., 2014).

### MRSA CC5 with sequence type 8

MRSA assigned to this clone are widespread and diverse across Africa as evidenced in 27 studies (Table 2). The first known early or ancestral clone, ST250-I [1B], was mainly associated with hospital-acquired infections in Ibadan, South-West Nigeria (Ghebremedhin et al., 2009), and recently observed in Ghana (Egyir et al., 2014b). ST8-II [2A] was only described in the KwaZulu-Natal region of South Africa (Essa et al., 2009), while a number of investigations reported ST8-IV [2B] in Angola (Conceição et al., 2014), Cameroon (Breurec et al., 2011b), Gabon (Schaumburg et al., 2011; Ateba Ngoa et al., 2012), Ghana (Egyir et al., 2014b), Madagascar (Breurec et al., 2011b), Nigeria (Adesida et al., 2005; Shittu et al., 2012), São Tomé and Príncipe (Conceição et al., 2013) and South Africa (Essa et al., 2009). The MRSA isolates from Angola possessed the SCC*mec* type IVd element (Conceição et al., 2014). ST612-IV [2B], a double locus variant (dlv) of ST8-IV [2B], and only recently reported as PVL-positive (Oosthuysen et al., 2014), is widespread across South Africa (Goering et al., 2008; Moodley et al., 2010; Jansen van Rensburg et al., 2011; Oosthuysen et al., 2014), alongside other variants such as ST1173/ST1338-IV [2B] (Shittu et al., 2009). The ST8-IV [2B] clone in South Africa was identified from a variety of clinical infections, in particular, bacteraemia, skin and soft tissue and wound infections (Shittu et al., 2009; Moodley et al., 2010; Jansen van Rensburg et al., 2011; Oosthuysen et al., 2014). An ST8-IVc [2B] strain (PVL-positive; *spa* type t062) was identified from a 4 day old male child with community-acquired invasive infection in Tunisia (Kechrid et al., 2011). Furthermore, ST8-V [5C] was described in Algeria (Antri et al., 2011), Angola (Conceição et al., 2014), Ghana (Egyir et al., 2013), Nigeria (Shittu et al., 2011, 2012; Raji et al., 2013), São Tomé and Príncipe (Conceição et al., 2013), and South Africa (Oosthuysen et al., 2014). Other STs observed within the CC5-ST8 cluster include ST8-SCC*mec-NT* in Gabon (Schaumburg et al., 2014) and Nigeria (Shittu et al., 2012), ST94-IV [2B] described in Nigeria (Shittu et al., 2011) and ST97-SCC*mec*-NT in Tunisia (Ben Jomàa-Jemili et al., 2013). In addition, ST247-I [1B] was reported only in Tunisia (Ben Jomàa-Jemili et al., 2013) and Nigeria (Raji et al., 2013), ST637-III [3A] in Algeria (Ramdani-bouguessa et al., 2006), ST1819-I [1B] in Tunisia (Ben Jomàa-Jemili et al., 2013), and ST2021-V [5C] in Ghana (Egyir et al., 2014b).

The “Brazilian/Hungarian clone” (ST239-III [3A]) is an hybrid of CC30 and CC8 based on a single large chromosomal replacement (Robinson and Enright, 2004), and ST241-III [3A] is a single locus variant (slv) of ST239-III [3A]. These two STs were identified concurrently in Algeria (Ramdani-bouguessa et al., 2006), Morocco, Niger and Senegal (Breurec et al., 2011b), and Tunisia (Ben Jomàa-Jemili et al., 2013). ST239-III [3A] has also been described in Ghana (Egyir et al., 2014b) and Kenya (Aiken et al., 2014), and consistently since 2001 in South Africa (Essa et al., 2009; Shittu et al., 2009; Moodley et al., 2010; Jansen van Rensburg et al., 2011; Oosthuysen et al., 2014). A recent study detected ST239 with the SCC*mec* type III_*mercury*_ [3A] in a tertiary health care facility in South-West Nigeria (Raji et al., 2013). ST241-III [3A] is the dominant clone in North-East Nigeria (Okon et al., 2009; Shittu et al., 2011, 2012). Interestingly, three SCC*mec* variants, ST239-IVa [2B], ST239/ST241-V [5C], and ST241-IV [2B], and associated with hospital-acquired infections were reported in Algeria (Ramdani-bouguessa et al., 2006), Niger (Breurec et al., 2011b), and Nigeria (Ghebremedhin et al., 2009).

### Clonal complex 22

In Africa, ST22 was identified only in Algeria (Ramdani-bouguessa et al., 2006), South Africa (Moodley et al., 2010; Jansen van Rensburg et al., 2011; Oosthuysen et al., 2014), and Tunisia (Ben Jomàa-Jemili et al., 2013). ST22-IV [2B] was related with hospital-associated infections in the Western Cape and KwaZulu-Natal provinces of South Africa. A variant of ST22 (ST22-V [5C]-PVL-positive) was also reported in an hospital in Western Cape, South Africa (Oosthuysen et al., 2014). The ST22 identified in Tunisia possessed a non-typeable SCC*mec* element (Ben Jomàa-Jemili et al., 2013). Besides, an ST636-SCC*mec*-NT isolate has also been reported in Algeria (Ramdani-bouguessa et al., 2006).

### Clonal complex 30

ST30-IVa [2B]-PVL-positive, also known as “South-West Pacific clone,” has been reported in Egypt (Enany et al., 2010), and a multicenter African study identified ST30-V [5C] only in Antananarivo, Madagascar (Breurec et al., 2011b). The hospital associated ST36-II [2A] (UK-EMRSA-16), was described only in South Africa (Goering et al., 2008; Moodley et al., 2010; Jansen van Rensburg et al., 2011; Oosthuysen et al., 2014), while ST39-II [2A] a dlv was identified in an hospital in Algiers, Algeria (Djoudi et al., 2013), and Ile-Ife, South-West Nigeria (Shittu et al., 2012). MRSA assigned with these groups (ST36-II [2A] and ST39-II [2A]) were PVL-negative. Furthermore, two SCC*mec* variants, ST37-IVa [2B] and ST37-III [3A], were reported in Algeria (Ramdani-bouguessa et al., 2006) and Nigeria (Shittu et al., 2012), respectively.

### Clonal complex 45

ST45-IV [2B], the “Berlin clone,” was detected in an hospital in the KwaZulu-Natal (South Africa) during a multicenter surveillance study (Essa et al., 2009) and ST45-V [5C] was reported in mother-infant pairs in Gabon (Schaumburg et al., 2014). An MRSA with a non-typeable SCC*mec* associated with community-acquired infections has been identified in Tunisia (Ben Jomàa-Jemili et al., 2013). Finally, ST508-V [5C], a slv to ST45, and also associated with community-acquired infections was described in Ghana (Egyir et al., 2014a).

### Clonal complex 80

The CC80 was limited to three North African countries: Algeria, Egypt, and Tunisia (Table 2). The European clone, ST80-IV [2B]-PVL-positive, was first described in Algeria from both hospitalized and outpatients (Ramdani-bouguessa et al., 2006), and has continued to be the leading clone in the country (Ramdani-bouguessa et al., 2006; Bekkhoucha et al., 2009; Antri et al., 2011; Djoudi et al., 2013). ST80-IVc [2B] has been identified in Egypt (Enany et al., 2010), and Tunisia (Ben Slama et al., 2011; Ben Jomàa-Jemili et al., 2013). In addition, sequence types related to ST80 have been recovered from human clinical samples (ST153-SCC*mec*-NT, ST728-IVc [2B], ST1440-IVc [2B], and ST2563-IVc [2B]) (Kechrid et al., 2011; Ben Jomàa-Jemili et al., 2013), and nasal specimen of healthy sheep (ST153-SCC*mec*-NT) (Gharsa et al., 2012) in Tunisia. The afore-mentioned sequence types, ST728, ST1440 and ST2563 belonged to accessory gene regulator (agr) type III and were PVL-positive. Moreover, a PVL-negative ST80-IVc [2B] with *agr* type II has also been detected in Tunisia (Ben Jomàa-Jemili et al., 2013), and a PVL-negative ST635-IV [2B] in Algeria (Ramdani-bouguessa et al., 2006).

### Clonal complex 88

The “West Australia MRSA-2 clone” (WA-MRSA-2), ST88-IV [2B], was reported in both hospital and community settings in eight African countries; Angola (Conceição et al., 2014), Cameroon (Breurec et al., 2011b), Gabon (Schaumburg et al., 2011; Ateba Ngoa et al., 2012), Ghana (Egyir et al., 2013, 2014b), Madagascar (Breurec et al., 2011b), Niger (Breurec et al., 2011b), Nigeria (Ghebremedhin et al., 2009; Shittu et al., 2012; Raji et al., 2013) and Senegal (Breurec et al., 2011b). The MRSA isolates from Angola possessed an SCC*mec* IVa [2B] element (Conceição et al., 2014). PVL-positive ST88-IV [2B] were detected in Nigeria (Ghebremedhin et al., 2009), and an SCC*mec* subtype ST88-IVa [2B] was identified among three health care workers and a patient in São Tomé and Príncipe (Conceição et al., 2013). The ST88-IV [2B] with *spa* type t3489 was also recovered from nasal samples of swine in Senegal (Fall et al., 2012). In addition, an SCC*mec* non-typeable ST88 was described from an out-patient in Algeria (Bekkhoucha et al., 2009), and a strain related to WA-MRSA-2 (ST1289-IV [2B]) was identified in Yaoundé, Cameroon (Breurec et al., 2011b).

### Other clonal complexes

CC7, CC121, and CC152 have been reported in Africa. The PVL-negative ST789 (assigned with CC7) was identified in Angola (with SCC*mec* IV [2B]) (Conceição et al., 2014). However, in Ghana, ST789 was PVL-positive and carried an SCC*mec* IV element [2B] (Egyir et al., 2014b). An ST1010-PVL-positive (CC121) with non-typeable SCC*mec* element has only been described in Egypt (Enany et al., 2010). Furthermore, PVL-positive MRSA assigned to CC152 (ST152-SCC*mec*-NT) was reported in Nigeria (Shittu et al., 2012).

## Discussion

MRSA has been reported in Africa, at least since 1978 (Scragg et al., 1978). This systematic review showed that adequate data on the molecular epidemiology of MRSA are limited, with reports from only 15 of the 54 African countries. No *spa* type was dominant, however, t042 and t044 were the major *spa* types identified in three North African countries (Table 2). Moreover, we did not observe a clear distinction between hospital- and community-associated MRSA clones in Africa which is in agreement with other investigations worldwide (Fossum Moen et al., 2013; Pasquale et al., 2013; Sherwood et al., 2013; Tavares et al., 2013). In this systematic review, the use of the current eBURST scheme grouped several African MRSA CCs (CC1, CC5, CC8, and CC7) into a single cluster, (CC5). This raises some concern on a suitable method for discrimination and grouping of *S. aureus* isolates. To overcome the above mentioned issue, whole genome sequencing approach might be the alternative (Dabul and Camargo, 2014).

Although a combination of factors could be responsible for the dissemination of clones between continents, increased movement of human population within or between countries might be one of the potential factors (Rogers et al., 2011). International travel could play a significant role in the transmission of MRSA, particularly the replacement of existing MRSA with fitter and more transmissible clones (Zhou et al., 2014). We observed that the predominant hospital-associated epidemic clones, EMRSA-15 [ST22-IV [2B]) and (EMRSA-16 [ST36-II [2A]), in the United Kingdom (UK) (Johnson et al., 2005) were reported only in South Africa (Goering et al., 2008; Moodley et al., 2010; Jansen van Rensburg et al., 2011; Oosthuysen et al., 2014). Moreover, ST80-IV [2B] (the European clone) has consistently been recognized as the predominant PVL-positive MRSA clone in North Africa (Ramdani-bouguessa et al., 2006; Bekkhoucha et al., 2009; Ben Nejma et al., 2009, 2013; Enany et al., 2010; Antri et al., 2011; Ben Slama et al., 2011; Ben Jomàa-Jemili et al., 2013; Djoudi et al., 2013). A recent report based on whole genome analysis provided strong evidence that the European ST80-IV [2B] was derived from a PVL-positive MSSA ancestor in sub-Saharan Africa that acquired the SCC*mec* IV element, and clonal spread was enhanced by increased transnational movement (Stegger et al., 2014). However, the factors responsible for the limited spread of the ST80-IV [2B] only in North Africa observed so far are unclear.

The SCC*mec* IV (and its subtypes) and SCC*mec* V were identified in several MRSA clones, and ST5 and ST8 clearly showed more diversity in terms of SCC*mec* types compared with other STs in Africa. The success of these SCC*mec* types (IV and V) could be due to their small sizes and low fitness costs (Enright et al., 2002; Okuma et al., 2002; Monecke et al., 2011). It is also noteworthy that the SCC*mec* types IVa and IVc were identified in genetically unrelated clones, e.g., ST5-IVa [2B] (CC5) in Algeria (Ramdani-bouguessa et al., 2006; Bekkhoucha et al., 2009), São Tomé and Príncipe (Conceição et al., 2013), ST8-IVc [2B] in Tunisia (CC5) (Kechrid et al., 2011), and ST37-IVa [2B] (CC30) in Algeria (Ramdani-bouguessa et al., 2006). This might suggest horizontal gene transfer or independent acquisition (Mašlaòová et al., 2013). Another interesting observation was the detection of the SCC*mec* type IVa and V in the hospital-associated ST239/ST241-III [3A] in Algeria (Ramdani-bouguessa et al., 2006), Nigeria (Ghebremedhin et al., 2009), and Niger (Breurec et al., 2011b). Since ancient MSSA strains for this ST have not been reported (Enright et al., 2002), our observation suggests that acquisition of these SCC*mec* types by MSSA is less likely, and points to the possible replacement of SCC*mec* type III with IV and V on the ST239/241 genome (Li et al., 2013).

Data on the epidemiology of MRSA in animals are limited in Africa (EL Seedy et al., 2012; Fall et al., 2012; Gharsa et al., 2012). Moreover, the genetic relatedness between human and animal MRSA has not been investigated (Table 2). It should be noted, however, that human-associated ST5-IV [2B], ST88-IV [2B], and ST153-SCC*mec-*NT have been reported from animals in Tunisia (Gharsa et al., 2012) and Senegal (Fall et al., 2012). Recently, human-associated *S. aureus* lineages were described in captive Chimpanzees in Gabon, Madagascar, Uganda and Zambia (Schaumburg et al., 2012, 2013; Nagel et al., 2013). Notably, a likely case of *S. aureus* transmission from a veterinarian to a chimpanzee from the same sanctuary was demonstrated (Schaumburg et al., 2012). Zoonotic transmission may constitute a major concern in Africa, where there is often substantial exposure to domesticated animals (Fall et al., 2012; Gharsa et al., 2012). Furthermore, animal-adapted clones might undergo further host-adaptive evolutionary changes, which could result in an epidemic spread of new and more virulent strains in the human population (Spoor et al., 2013). Other risk factors for animal to human MRSA transmission, which include contaminated environment (Verkade and Kluytmans, 2013) and meat products (Hamid and Youssef, 2013), have not been investigated in Africa. Livestock-associated MRSA are widespread in Europe, but the transmission of these strains to humans is either rare or limited to people with direct contact with MRSA infected/carrier animals (Verkade and Kluytmans, 2013). Using whole genome sequencing, evidence of zoonotic transmission of MRSA harboring *mecC* was reported in Denmark (Harrison et al., 2013). The *mecC*-positive MRSA, initially known as a livestock MRSA belonging to the CC130, is recognized in both animals and humans in Europe (Paterson et al., 2014a). In addition, this clone has been implicated in severe infections in humans (Paterson et al., 2014b), resulting in one death (García-Garrote et al., 2014). The clinical importance of *mecC*-positive MRSA is not yet clear in Africa as data is unavailable. Therefore, we suggest that surveillance for MRSA should include detection of the *mecC* gene where *mecA* is not detected in resistant isolates.

This systematic review did not seek to provide comprehensive information on the burden of PVL-positive MRSA in Africa. However, it provided some interesting observations on their epidemiology in Africa such as the identification of PVL-positive isolates assigned with CC7 (originally classified with CC152) in Ghana (Egyir et al., 2014a), CC88-IV [2B] in Cameroon (Breurec et al., 2011b) and Nigeria (Ghebremedhin et al., 2009), ST612-IV [2B] in South Africa (Oosthuysen et al., 2014), and CC152 in Nigeria (Shittu et al., 2012). Until now, CC152 was only described in the Balkan region (Francois et al., 2008). The mode of acquisition of the *mecA* gene by ST152 is still unknown, but it might be explained by either its introduction through international travel or the acquisition of the methicillin resistance gene by PVL-positive MSSA, which is prevalent in West and Central Africa (Ruimy et al., 2008; Okon et al., 2009; Breurec et al., 2011a; Schaumburg et al., 2011; Shittu et al., 2011, 2012; Egyir et al., 2014a). These observations highlight the need for further surveillance data (including information on community-acquired necrotizing pneumonia) to understand the epidemiology of PVL-associated *S. aureus* in both hospital and community settings on the African continent.

## Conclusion

A number of pandemic MRSA clones were identified in Africa. In contrast, some MRSA clones are limited to specific countries or regions. Although the eBURST snapshot provided a description of the relationship between the MRSA clones reported in Africa and other lineages submitted into the MLST database from other continents, the objective of this review was not to understand the origin of MRSA clones in Africa, as this will require in depth analysis like whole genome sequencing. However, it did show that CC5 is the largest group and predominant in Africa. Nevertheless, the limited data available on MRSA in Africa draw attention to the need for increased surveillance of MRSA and molecular epidemiological studies. We strongly recommend improved co-operation between clinicians and microbiologists in Africa. This synergy could provide an understanding on the local epidemiology of MRSA. In addition, we strongly advocate the establishment of effective diagnostic microbiology facilities that will incorporate high-throughput technologies for monitoring the clonal expansion and dissemination of MRSA. In the meantime, increased networking through collaboration with *S. aureus* reference centers could provide support for genotyping services to African countries with limited resources. Finally, population-based surveillances for MRSA are needed to evaluate the situation of community associated MRSA as well as studies on MRSA from animal hosts. To understand the origin of the newly emerged clones, MSSA genotyping needs to be incorporated with MRSA surveillance studies.

## Author contributions

MK, AS, and SMA initiated the project. SMA extracted the data and reviewed the articles with MK. SMA, AS, MN, and MK wrote the manuscript. All the authors reviewed the final version of the manuscript prior to submission for publication

## Financial support

This systematic review was supported by the Organization for Women in Science for the Developing World; Clinical Infectious Diseases Research Initiative, University of Cape Town, South Africa; Bill and Melinda Gates Foundation Global Health Grant (OPP107641), United States of America; Deutscher Akademischer Austausch Dienst, Germany; Carnegie Corporation of New York, United States of America. Any opinions, findings and conclusions, or recommendations expressed in this review are those of the authors, and therefore do not represent the official position of the funders.

### Conflict of interest statement

The authors have no conflict of interest to declare related to the content of this paper. The funders had no role in the study design, data collection and analysis, decision to publish, or preparation of the manuscript. The first and corresponding authors had full access to the study data. All authors had final responsibility for the decision to submit the article for publication. The authors declare that the research was conducted in the absence of any commercial or financial relationships that could be construed as a potential conflict of interest.

## Footnotes

^1^eBURST [Online]. Available online at: http://www.mlst.net version 3.
