# Supplementary material for: Molecular epidemiology of Methicillin-resistant Staphylococcus aureus in Africa: a systematic review
Source: Front Microbiol. 2015 Apr 30;6:348. doi: 10.3389/fmicb.2015.00348 (PMC4415431; doi:10.3389/fmicb.2015.00348)
Supplement: Supplementary file 1 [file Table1.PDF]

Table S1: Keywords used to identify eligible studies available in four biomedical databases

Database	Search period	Search strategy
MEDLINE via PubMed	1974 - October 2014	(MRSA OR Methicillin-resistant Staphylococcus aureus)<br>**AND**<br>(Algeria OR Angola OR Benin OR Botswana OR Burkina Faso OR “Burkina Faso” OR Burkina Fasso OR Upper Volta OR "Upper Volta" OR Burundi OR Cameroon OR Cape Verde OR "Cape Verde" OR Central African Republic OR Chad OR Comoros OR “Iles Comores” OR Iles Comores OR Comoro Islands OR “Comoro Islands” OR Congo OR Democratic Republic Congo OR “Democratic Republic of the Congo” OR Zaire OR Djibouti OR Egypt OR Equatorial Guinea OR "Equatorial Guinea" OR Eritrea OR Ethiopia OR Gabon OR Gambia OR Ghana OR Guinea OR Guinea Bissau OR "Guinea Bissau" OR Ivory Coast OR “Ivory Coast” OR Cote d'Ivoire OR "Cote d'Ivoire" OR Kenya OR Lesotho OR Liberia OR Libya OR Libia OR Jamahiriya OR Jamahiryia OR Madagascar OR Malawi OR Mali OR Mauritania OR Mauritius OR Ile Maurice OR “Ile Maurice” OR Morocco OR Mozambique OR Moçambique OR Namibia OR Niger OR Nigeria OR Rwanda OR Sao Tome OR "Sao Tome" OR Senegal OR Seychelles OR Sierra Leone OR "Sierra Leone" OR Somalia OR South Africa OR "South Africa" OR Sudan OR South Sudan OR “South Sudan” OR Swaziland OR Tanzania OR Tanganyika OR Zanzibar OR Togo OR Tunisia OR Uganda OR Western Sahara OR "Western Sahara" OR Zambia OR Zimbabwe OR Africa OR Africa* OR Southern Africa OR West Africa OR Western Africa OR Eastern Africa OR East Africa OR North Africa OR Northern Africa OR Central Africa OR Sub Saharan Africa OR Subsaharan Africa OR Sub-Saharan Africa)<br>NOT<br>(Guinea pig* OR "Guinea pig*" OR Aspergillus niger OR "Aspergillus niger" OR Europe* OR America* OR Asia*)
EBSCOhost via Academic Search premier, Africa-Wide information and CINAHL	1982 - October 2014	
ISI web of knowledge	1950 – October 2014	
Scopus from SciVerse	1982 – October 2014	(MRSA OR methicillin-resistant staphylococcus aureus)<br>**AND**<br>(Africa)¶

¶ In order to exclude studies from other continents, the African countries was manually selected as recommended by Scopus database.
